# Fatigue is common and severe in patients with mastocytosis

**DOI:** 10.1177/2058738418803252

**Published:** 2018-10-23

**Authors:** Roald Omdal, Inger Marie Skoie, Tore Grimstad

**Affiliations:** 1 Clinical Immunology Unit, Department of Internal Medicine, Stavanger University Hospital, Stavanger, Norway; 2 Department of Clinical Science, Faculty of Medicine, University of Bergen, Bergen, Norway; 3 Department of Dermatology, Stavanger University Hospital, Stavanger, Norway; 4 Unit of Gastroenterology, Department of Internal Medicine, Stavanger University Hospital, Stavanger, Norway

**Keywords:** brain, fatigue, innate immunity, mastocytosis

## Abstract

Chronic fatigue is a common phenomenon in inflammatory and autoimmune conditions, in cancer, and in neurodegenerative diseases. Although pain and psychological factors influence fatigue, there is an increasing understanding that there is a genetic basis, and that activation of the innate immune system is an essential generator of fatigue. Mast cells are important actors in innate immunity and serve specialized defense responses against parasites and other pathogens. They are also major effector cells in allergic reactions. Primary disorders causing constitutively hyperactivity of mast cells are called mastocytosis and are frequently due to a gain-of-function mutation of the *KIT* gene encoding the transmembrane tyrosine kinase receptor. It is a clinical experience that patients with mast cell disorders suffer from fatigue, but there is a lack of scientific literature on the phenomenon. We performed a controlled study of fatigue in mastocytosis patients and document a 54% prevalence of clinical significant fatigue.

## Introduction

*Mastocytosis* is a term that encompasses the primary mast cell (MC) disorders and is divided into a systemic form, a cutaneous form, and the rare MC sarcoma. MCs develop from myeloid stem cells in response to stimulation by stem-cell factor and migrate from the blood into various tissues where they mature and acquire specific phenotypes influenced by the local environment. The majority of patients with mastocytosis display a gain-of-function mutation of the *KIT* gene that encodes the transmembrane tyrosine kinase receptor (CD117), and this renders MCs constitutively hyperactive. A variety of symptoms and signs follow the continuous degranulation and release of histamine, tryptase, serotonin, pro-inflammatory cytokines, and other biological mediators from MCs and give rise to cardiovascular, cutaneous, digestive, musculoskeletal, neurologic, respiratory, and systemic phenomena.^1^

It is a clinical experience that patients with mastocytosis suffer from severe fatigue and may report worsening of fatigue hours to days before outbreak of disease attacks. To our knowledge, only one case report has enlightened this issue of the mastocytosis symptom spectrum.^2^ On the other side, cognitive disturbances and cerebral involvement are acknowledged, but the exact pathophysiology remains obscure.^3^ Although much debated and thought to have multifactorial origin, emerging evidence points to a genetic and molecular basis for fatigue.^4^ Fatigue is generated at least partly through innate immunity responses, and MCs are strong activators of innate immunity. It is therefore to be expected that fatigue is a significant complaint among patients with MC disorders, but there is a lack of literature based on systematic studies regarding this issue, as far as we can understand. We recently had the opportunity to investigate 28 subjects with mastocytosis, rate their fatigue, and compare findings with healthy subjects.

## Subjects and methods

Twenty-eight patients with mastocytosis attending a national educational meeting were investigated. In addition, 28 healthy control subjects matched for age (±5 years) and gender were selected from our research cohorts on fatigue (Table 1). The severity of fatigue was rated by the fatigue Visual Analog Scale (fVAS), a generic instrument that is widely used to measure fatigue in various diseases.^5^ It consists of a 100 mm horizontal line with wording “no fatigue” at the left anchor and “fatigue as bad as it can be” at the right anchor. A higher score indicates a more fatigue, and an fVAS score >50 is often regarded as clinical significant fatigue.^6^

**Table 1.** Descriptive data in 28 mastocytosis patients and 28 healthy control subjects.

Variable	Patients (n = 28)	Healthy subjects (n = 28)	*P*
Age, years	51 (21–72)	53 (21–71)	0.33
Gender, females no. (%)	22 (78.6)	22 (78.6)	1.00
fVAS, scores	53 (15–91)	6 (0–35)	<0.001

fVAS: fatigue Visual Analog Scale.

Medians (ranges) are given except for gender.

### Statistical analysis

Normality of data was tested with the Shapiro–Wilk test. Some data were not normality distributed and the results are thus presented as median and ranges for continuous data and as counts and percentages for categorical data. The Wilcoxon signed-rank test was used to compare two groups of continuous data.

### Ethics

This study was carried out in compliance with the Helsinki Declaration and approved by the Regional Committee for Medical and Health Research, West (2010/1455; 2011/2631). All subjects gave informed consent to participate in the study.

## Results

**Figure 1.**
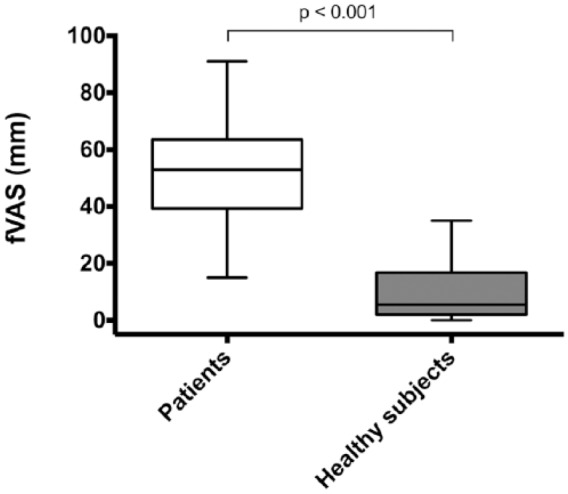
Fatigue in 28 patients with mastocytosis compared with 28 age- and gender-matched healthy subjects. fVAS: fatigue Visual Analog Scale.

Patients with mastocytosis reported a median fVAS score of 53 (15–91) versus 6 (0–35) in the healthy subjects; *P* < 0.001 (Figure 1). If subjects were categorized in clinical significant fatigue versus not significant fatigue (fVAS score ⩾50 vs <50), 13 out of the 28 patients (54%) had fatigue, while none of the healthy subjects reported fatigue. Fatigue scores were not associated with age or gender in either group.

## Discussion

This observation indicates that fatigue is a prevalent and clinically significant phenomenon in about half of patients with mastocytosis. The reported prevalence and severity match with findings in other chronic inflammatory conditions in which this issue has been more systematically investigated.^6,7^

Fatigue is increasingly being recognized as a prominent and severe phenomenon of chronic inflammatory and autoimmune diseases, cancer, and various other chronic conditions. Although the pathophysiology is much debated, a conceptual biological model for understanding fatigue is the sickness behavior response, an evolutionary strongly based phenomenon triggered by innate immunity activation to invading pathogens and damage.^8^ This unconscious and automated response is characterized by sleepiness, depressive mood, social withdrawal, and loss of grooming, thirst, appetite, and initiative and is supposed to increase survival of the sick animal. Fatigue is a dominant feature of this response. Several animal studies have demonstrated the fundamental role pro-inflammatory cytokines, especially interleukin (IL)-1β, play in this response.^8^ In conditions with infection and/or tissue injury, activation of innate immunity cells will rapidly lead to increased production of IL-1β which pass through the blood–brain barrier (BBB) and reaches neuronal cells in the brain by both passive and active transport systems and can even be produced intrathecally. Once in the brain, IL-1β binds to a subtype of the IL-1 receptor and to a brain isoform of the accessory protein, the IL-1RaAcPb.^9^ Thus, while IL-1β in the periphery is a strong inducer of innate immunity-based inflammation, IL-1β directly modulates synaptic transmission through neuronal potassium and calcium influx (without inflammation) in the brain and induces subconscious and irresistible sickness behavior. In chronic inflammatory diseases, these processes are continuously active and sickness behavior (and fatigue) becomes chronic. Increased activation of IL-1β in the brain is observed in human subjects with chronic inflammatory and autoimmune conditions and severe fatigue,^10^ and treatment with IL-1 blocking agents alleviates fatigue.^11^

MCs serve important functions in innate immunity surveillance and carry out specialized defense responses against parasites and other pathogens when TLRs or G protein-coupled receptors are activated by peptidoglycans, snake venoms, wasp toxins, and so on. MCs are also major effector cells of allergic reactions. Whatever the primary response, degranulation of MCs releases a vast number of biological active molecules involved in innate immunity responses resulting in a focused and optimal attack on the invading pathogen.

**Figure 2.**
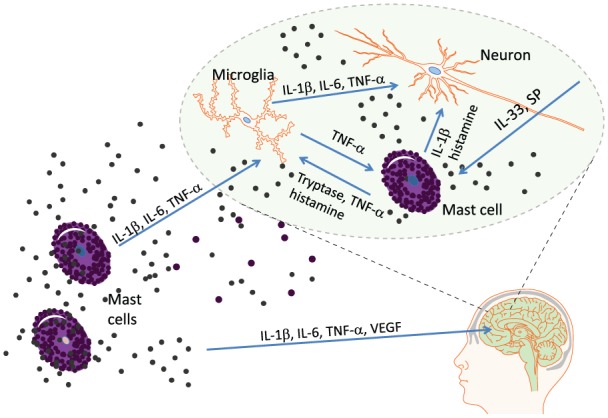
Proposed model for generation of fatigue in mastocytosis. MCs both in the periphery and in the brain produce and secrete pro-inflammatory cytokines, histamine, proteases, substance P, and other highly active signaling and reactive substances. VEGF disrupts the blood–brain barrier and augments influx to the brain of immune cells, cytokines, and other signaling molecules. Activated microglia and MCs secrete IL-β that bind to specific IL-1 receptors on cerebral neurons and induce the sickness behavior response, in which fatigue is a major element. VEGF: vascular endothelial growth factor; SP: substance P.

A hypothetical model for generation of fatigue in mastocytosis is therefore that activated MCs outside the brain release IL-1β, IL-6, TNF-α, and other bioactive molecules that pass the BBB and activate neuronal cells as well as microglia (Figure 2). Substance P (SP) and IL-33 together markedly enhance the production and release of TNF-α in MCs and leads to an increase in other pro-inflammatory cytokines.^12^ Vascular endothelial growth factor (VEGF) disrupts the BBB and augments trafficking of immune cells and signaling substances across the BBB. Inside the brain, activated MCs secrete tryptase, histamine, IL-1β, and TNF-α that trigger microglial cells to produce IL-1β which bind to adjacent brain-specific neuronal IL-1 receptors. Activation of these receptors induces sickness behavior.

### Weaknesses of the study

The patients were investigated during a national educational meeting for patients with different MC disorders. We had no access to the exact subtype of disorders, nor to tryptase levels, IgE-mediated allergies or other comorbidities. These matters obviously influence the interpretation of the results. Nevertheless, we think that the study throw light on a phenomenon that has gained relatively little attention in patients with MC disorders. Also, use of H1-antihistamines and sleep disorders due to nocturnal itch are phenomena that may influence fatigue experience and should be included in future studies.

In conclusion, our observation emphasize that fatigue is a prevalent and significant clinical phenomenon of the mastocytosis disease spectrum and can be explained in a biological context as part of the sickness behavior response driven by innate immunity mechanisms.
